# Right ventricular myocardial energetic model for evaluating right heart function in pulmonary arterial hypertension

**DOI:** 10.14814/phy2.15136

**Published:** 2022-05-18

**Authors:** Jacqueline V. Scott, Tanuf U. Tembulkar, Meng‐Lin Lee, Bradley T. Faliks, Kelly L. Koch, Anton Vonk‐Nordegraaf, Keith E. Cook

**Affiliations:** ^1^ Department of Biomedical Engineering Carnegie Mellon University Pittsburgh Pennsylvania USA; ^2^ Division of Cardiovascular Surgery Department of Surgery Cathay General Hospital Taipei Taiwan; ^3^ Department of Surgery University of Michigan Ann Arbor Michigan USA; ^4^ Department of Pulmonary Medicine Amsterdam University Medical Centers Vrije Universiteit Amsterdam Amsterdam the Netherlands

**Keywords:** pulmonary arterial hypertension, right heart failure, right ventricular energetics

## Abstract

**Background:**

Pulmonary arterial hypertension (PAH) increases right ventricular (RV) workload and decreases myocardial oxygen reserve, eventually leading to poor cardiac output. This study created and assessed a novel model of RV work output based on RV hemodynamics and oxygen supply, allowing new insight into causal mechanisms of RV dysfunction.

**Methods:**

The RV function model was built upon an earlier, left ventricular model and further adjusted for more accurate clinical use. The model assumes that RV total power output (1) is the sum of isovolumic and stroke power and (2) is linearly related to its right coronary artery oxygen supply. Thus, when right coronary artery flow is limited or isovolumic power is elevated, less energy is available for producing cardiac output. The original and adjusted models were validated via data from patients with idiopathic PAH (*n* = 14) and large animals (*n *= 6) that underwent acute pulmonary banding with or without hypoxia.

**Results:**

Both models demonstrated strong, significant correlations between RV oxygen consumption rate and RV total power output for PAH patients (original model, *R* ^2^ = 0.66; adjusted model, *R* ^2^ = 0.78) and sheep (original, *R* ^2^ = 0.85; adjusted, *R* ^2^ = 0.86). Furthermore, the models demonstrate a significant inverse relationship between required oxygen consumption and RV efficiency (stroke power/total power) (*p *< 0.001). Lastly, higher NYHA class was indicative of lower RV efficiency and higher oxygen consumption (*p* = 0.013).

**Conclusion:**

Right ventricular total power output can be accurately estimated directly from pulmonary hemodynamics and right coronary perfusion during PAH. This model highlights the increased vulnerability of PAH patients with compromised right coronary flow coupled with high afterload.

## Graphical abstract

This work presents, for the first time, an accurate model of right ventricular (RV) total work under high afterload as determined by thermodynamics. The model demonstrates a clear phenotypic difference in RV efficiency and aerobic metabolism between patients of mild and severe heart failure symptoms. Such a model could provide new goals for improving RV function in PAH patients.

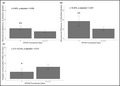

## 1 INTRODUCTION

Right ventricular (RV) function is the primary determinant of survival in patients with pulmonary arterial hypertension (PAH) (Rosenkranz et al., 2020). Abnormal increases in right ventricular afterload lead to eventual decreases in cardiac output and increased venous congestion, a vicious cycle that further strains the right ventricle and ends in right ventricular failure.

Per the 2015 European Respiratory Society guidelines, clinicians are instructed to focus directly on metrics of RV output (RV ejection fraction, cardiac output, stroke volume, etc.) as markers of disease severity and RV adaptation (Galiè et al., 2015). These metrics are crucial; however, less consideration is given to how RV output is a direct function of RV metabolic substrate delivery, particularly the oxygen required to power ATP production to fuel RV contraction (Ryan & Archer, 2014). As PAH progresses, multiple factors can limit right coronary perfusion including increased RV systolic pressure that drastically reduces right coronary systolic flow and reduction in cardiac output that reduces the driving pressure for coronary perfusion (Wolferen et al., 2008). Further, the estimated prevalence of coronary artery disease in PAH patients is 28%, a condition that further limits coronary perfusion (Shimony et al., 2011). The culmination of these factors leads to a reduction in total oxygen delivered to the right ventricle. Reduced right coronary perfusion reserve has been observed in PAH patients (Vogel‐Claussen et al., 2011). Once right ventricular oxygen extraction and coronary flow reserves are exhausted, patients will be less capable of tolerating a new physical insult to their cardiovascular system, such as physical exertion.

The RV requires high oxygen delivery to overcome the high afterload, but it must also be able to use this oxygen efficiently. Myocardial external efficiency, the observed ratio of stroke work to RV myocardial oxygen consumption, has been shown to be higher in PAH patients of New York Heart Association Class II (NYHA II) versus Class III/IV, linking inefficient oxygen use to disease severity (Wong, Ruiter, et al., 2011). However, it is unclear if this inefficiency reflects higher RV load, a preferential switch to aerobic glycolysis over oxidative phosphorylation, or one of the other possible causes that have been identified, including but not limited to septal bowing, neurohumoral metabolism, and increased heart rate. A statistical model published in 2011 by Wong et al. suggested that the main predictors of RV myocardial oxygen consumption (MVO_2_) in patients with PAH were heart rate and systolic pulmonary arterial pressure but did not provide the specific physiological mechanism by which these values determine MVO_2_ (Wong, Westerhof, et al., 2011). Pressure‐volume area (PVA) models of ventricular energetics have been applied to other heart failure modalities but only represent a fraction of oxygen consumption (roughly 40%), are difficult to measure reliably, and are more invasive as they require manual manipulation of venous return (Elbeery et al., 1995; Suga et al., 1981). Further, they do not reflect true thermodynamic first principles of energy expenditure under isovolumic contraction (Elbeery et al., 1995). Although pressure‐volume loops are not currently used to assess PAH patients, a more accurate energetic model of the RV under high afterload could prove valuable to assessment of PAH RV dysfunction and guiding optimization of extracorporeal membrane oxygenation, which can be used as a bridge‐to‐transplant (Galiè et al., 2015).

In order to improve understanding of how right ventricular oxygen demand and efficiency changes due to PAH, a novel right ventricular work model is proposed that builds upon the earlier work of Elbeery et al. (1995) on the left ventricle. The underlying premise of this model is that (1) ventricular total work capacity is dictated primarily by its oxygen supply and that (2) energy wasted due to high “isovolumic” work demands results in less energy available to produce a sufficient cardiac output. Here, the model is modified to more accurately portray the relationship between right ventricular hemodynamics and oxygen consumption, resulting in the first model to use thermodynamic first principles to calculate right ventricular power output specific to PAH. The accuracy of the original and adjusted models was then examined using data from clinical studies of patients with idiopathic PAH and from large animal models that underwent a combination of acute pulmonary hypertension with or without hypoxia. Finally, the clinical relevance of this model for improving assessment of RV function is thoroughly discussed.

## 2 METHODS

### 2.1 Clinical versus animal study model differences

Due to discrepancies in available instrumentation and avoidance of invasive measures in clinical studies, some modeling approaches differ between animal study data and clinical study data. A summary of differences is provided in Table 1 and discussed in more detail in the section below.

**TABLE 1:** Summary of differences between modeling approach for clinical studies and animal studies

	Clinical studies (*N* = 15)	Animal studies (*N* = 6)
Cause of increased pulmonary impedance	Idiopathic	Pulmonary Arterial Banding
Right coronary flow measurement	PET Imaging	Ultrasonic flow probe
Pulmonary arterial flow measurement	Estimated with invented flow profile	Ultrasonic flow probe
Right ventricular oxygen extraction fraction measurement	PET Imaging	Estimated from literature values

### 2.2 Metabolic right ventricular function model

The model presented here builds upon the model of Elbeery et al. (1995), originally designed and validated in canine left ventricles. Changes are made to make the model more appropriate for PAH pathophysiology, RV function, and human clinical studies.

In a normal adult human heart, oxidative phosphorylation is responsible for almost all ATP generation in myocardial tissue (95%), with glycolysis providing the remaining percentage (Martínez et al., 2017). While typically the work demands on the RV are low and oxygen supply is abundant, in situations of high afterload, there is significant evidence that RV work is limited by RV oxygen supply, similar to that of a healthy left ventricle (Vogel‐Claussen et al., 2011; Wong, Westerhof, et al., 2011). This model, therefore, uses this assumption to couple RV oxygen consumption directly to its energy expenditure via the following equation:

(1) $$\text{Total Mechanical Energy}\;\left(J\right)=20.2\times O_{2}\;\text{Consumption,}$$

where O_2_ Consumption is in ml per heartbeat and the constant factor is based on the theoretical efficiency of oxidative phosphorylation with mixed substrates (i.e., 20.2 joules per ml of oxygen) (Martínez et al., 2017). The units for Equation 1 are joules (J) per heartbeat. In this equation, the small contribution of anaerobic energy sources (glycolysis) is considered to be negligible (see Discussion for further comment).

The rate of oxygen consumption can, therefore, be coupled to the power output of the RV, or work per unit time.

(2) $$\begin{array}{c} 20.2\times O_{2}\;\text{Consumption Rate}\;\left(\frac{J}{\min}\right)=\\ \;\text{Total Mechanical Energy}\times \text{HR}, \end{array}$$

where HR is the heart rate (beats/min) and O_2_ Consumption Rate is in volume per min (ml/min). The units for Equation 2 are joules per minute (J/min).

The rate of O_2_ delivery to the right ventricle free wall is determined primarily by right coronary flow in humans (Crystal & Pagel, 2018). In sheep, there is anatomical evidence that the left anterior descending branch of the left coronary artery also supplies blood to the upper region of the right ventricle, but its contribution has not been quantified, and this model assumes its effect on right ventricular oxygen supply to be negligible (Likitha et al.). This relationship is modeled as:

(3) $$O_{2}\;\text{Delivery Rate}\;\left(\frac{\text{ml}}{\min}\right)=1.34\times \text{OEF}\times Q_{\text{RCA}}\times S_{a}\times C_{\text{hb}},$$

where *S*_a_ is the arterial oxygen saturation (%), OEF is the RV oxygen extraction fraction, *Q*_RCA_ is the right coronary flow (ml/min), *C*_hb_ is the concentration of hemoglobin (g/ml), and the constant (1.34) is the hemoglobin oxygen carrying capacity (1.34 ml of O_2_ per gram of hemoglobin) (Cotton et al., 2015). The units for Equation 3 are milliliters per minute (ml/min). To avoid invasively measuring arteriovenous differences in oxygen saturation during acute sheep studies, RV OEF was not directly measured, but instead estimated to be 50% in normoxic and 60% in hypoxic conditions (Hart et al., 2001; Saito et al., 1991). RV OEF for clinical studies, however, was determined directly through PET imaging (Lubberink et al., 2011; Wong, Ruiter, et al., 2011).

According to the Elbeery et al model, total mechanical energy (TME), in joules (J), is dictated by both an internal index of heat (referred to here as isovolumic work) and stroke work (Elbeery et al., 1995):

(4) TME(J)=Isovolumic Work+Stroke Work

The units for Equation 4 are joules (J). RV total power output (TPO), in joules per minute (J/min), is therefore the sum of isovolumic work and stroke work over a cardiac cycle:

(5) $$\text{TPO}\;\left(\frac{J}{\min}\right)=\text{HR}\times \left(\text{Isovolumic Work}+\text{Stroke Work}\right),$$

The units for Equation 5 are joules per minute (J/min).

In the Elbeery et al model, isovolumic power is estimated via:

(6) $$\begin{array}{c} \text{Isovolumic Power}\;\left(J/\min\right)=\text{HR}\times {\text{mRV}}_{\text{EDV}}\times \\ \;({\text{RV}}_{\text{EDV}}‐V_{0})\times \left(1.33\times 10^{‐4}\right), \end{array}$$

where mRV_EP_ is the mean right ventricular ejection pressure (mmHg) and RV_EDV_ is the right ventricular end‐diastolic volume during systole (Elbeery et al., 1995). The units for Equation 6 are joules per minute (J/min). The constant (1.33 × 10^−4^) is the conversion factor from mmHg‐ml to joules, and *V*_0_, is the dead volume that is determined by the ventricular end‐systolic pressure‐volume relationship, is considered to be negligible (Elbeery et al., 1995).

Ventricular stroke work is calculated as:

(7) $$\text{Stroke work}\;\left(J/\min\right)=\int_{0}^{T}P_{\text{PA}}Q_{\text{PA}},$$

where *P*_PA_ and *Q*_PA_ are the instantaneous pulmonary arterial pressure and flow measurements, respectively, and *T* is the period of RV ejection. The units for Equation 7 are joules per minute (J/min).

Although *Q*_PA_ could be directly measured in the acute sheep studies via flow probe, this cannot be done for clinical patients and pulmonary arterial flow was instead approximated using an invented flow profile (Grondelle, 1982). The invented flow profile was based on the following piecewise function:

(8) $$\begin{array}{c} Q_{i}\left(\text{ml}/\min\right)=\\ \left\{\begin{array}{cc} A\left(\text{SV}\right)\exp\left(‐3t\right)\sin\left(\frac{\pi t}{t_{\text{es}}}\right) & 0<t<t_{\text{es}}+0.02\\ Q_{i}\left(t_{\text{es}}+0.02\right)\left[1‐\frac{t‐t_{\text{es}}‐0.02}{0.02}\right] & t_{\text{es}}+0.02<t<t_{\text{es}}+0.04\\ 0 & t_{\text{es}}+0.04<t<t_{\text{ed}} \end{array}\right. \end{array}$$

where *A* is an amplitude proportionality factor, SV is the stroke volume in ml, *t*_es_ is the time at which RV ejection ends in seconds, *t*_ed_ is the time at which RV diastole ends in seconds, and 0.04s is assumed to be the duration of pulmonary valve regurgitation. The units for Equation 8 are milliliters per minute (ml/min). The value of the amplitude proportionality factor *A* was calibrated such that mean *Q_i_* (*t*) was equivalent to each patient's respective cardiac output. Finally, the invented flow (*Q_i_* (*t*)) and discrete pulmonary arterial pressure data were integrated for the first five sequential heartbeats during the period of ejection using the trapezoidal method to calculate stroke work. The stroke work for each heartbeat was divided by the period of ejection (*T*) to obtain stroke power.

(9) $$\text{Stroke work}\;\left(J/\min\right)=\frac{1}{T}\int_{0}^{T}P_{\text{PA}}Q_{\text{PA}},$$

The units for Equation 9 are joules per minute (J/min). Stroke power is averaged over five heartbeats to get a steady‐state measurement at rest. Details on how the period of ejection was determined in the RV pressure waveform can be found in the Supporting Information.

The following adjusted model was compared to the Elbeery et al. (1995) model:

1. Assume that isovolumic work is proportional to raising the RV end‐systolic volume (RV_ESV_) to the mean ejection pressure, rather than the end‐diastolic volume (RV_EDV_). This provides a lower bound for total RV work, by assuming that the majority of work is performed in raising the entire end‐diastolic volume to the mean ejection pressure and that stroke volume ejection is a passive consequence of the resulting pressure gradient between the RV and distal PA.
2. Assume that isovolumic work is proportional to the difference between mean ejection pressure (mRV_EP_) and end‐diastolic pressure (RV_EDP_), rather than mean ejection pressure alone. Arguably, initially raising the right ventricle to its end‐diastolic pressure is work performed by the left ventricle. In patients with PAH, RV_EDP_ can be significantly raised due to venous congestion, making this change crucial to model accuracy.

With the modifications above, the adjusted model changes Equation 6 to be:

(10) $$\begin{array}{c} \text{Isovolumic Power}\;\left(J/\min\right)=\text{HR}\times \left({\text{mRV}}_{\text{EP}}‐{\text{RV}}_{\text{EDP}}\right)\times \\ \;({\text{RV}}_{\text{ESV}})\times \left(1.33\times 10^{‐4}\right), \end{array}$$

The units for Equation 10 are joules per minute (J/min).

- In humans, zeroth harmonic stroke power (the product of mean pulmonary arterial pressure and cardiac output) can be calculated directly. Out of necessity, the non‐zeroth harmonic stroke power is estimated using the method of Saouti et al. (2010), by which oscillatory work is estimated to be proportional to pulmonary arterial pulse pressure. Stroke work is therefore estimated in the adjusted model by:

(11) $$\begin{array}{c} \text{Stroke work}\;\left(J/\min\right)=\text{HR}\times \left(\text{mPAP}\right)\times \left(\text{SV}\right)\times \\ \;\left(1.33\times 10^{‐4}\right)+\left(0.156\times \text{PP}\right) \end{array}$$

The units for Equation 11 are joules per min (J/min).

As there is no known equivalent study performed in sheep to relate the pulmonary arterial pulse pressure and oscillatory power, the method for calculating stroke work in Equation 9 is used when applying the adjusted model to animal data. Provided that the instantaneous *Q*_PA_ is measured and not approximated, this equation is the truest measure of total stroke work and Equation 11 is only used as a stopgap to estimate pulsatile work without the ability to measure *Q*_PA_ in humans.

Total power output for the Elbeery et al model is then:

(12) $$\begin{array}{c} \text{TPO}\;\left(\frac{J}{\min}\right)=\text{HR}\times {\text{mRV}}_{\text{EP}}\times \left({\text{RV}}_{\text{EDV}}‐V_{0}\right)\times \\ \;\left(1.33\times 10^{‐4}\right)+\frac{1}{T}\int_{0}^{T}P_{\text{PA}}*Q_{\text{PA}}, \end{array}$$

which is the sum of Equations 6 and 9 (Elbeery et al., 1995). The units for Equation 12 are joules per minute (J/min)

This is contrasted with total power output for the adjusted model:

(13) $$\begin{array}{c} \text{TPO}\;\left(J/\min\right)=\text{HR}\times \left({\text{mRV}}_{\text{EP}}‐{\text{RV}}_{\text{EDP}}\right)\times \left({\text{RV}}_{\text{ESV}}\right)\times \\ \left(1.33\times 10^{‐4}\right)+\text{HR}\times \left(\text{mPAP}\right)\times \left(\text{SV}\right)\times \left(1.33\times 10^{‐4}\right)\\ +\left(0.156\times \text{PP}\right) \end{array}$$

which is the sum of Equaions 10 and 11. The units for Equation 13 are joules per minute (J/min).

Our final RV efficiency metric is then:

(14) $$\text{RV Efficiency}\;\left(\%\right)=\frac{\text{Useful Stroke Power}}{\text{Total Power Output}}$$

where Useful Stroke Power (J/min) is defined as non‐pulsatile stroke power, in order to differentiate between stroke power that represents forward flow into the pulmonary artery versus stroke power loss to pulsatile effects that does not generate forward blood flow.

(15) Useful Stroke Power(J/min)=mPAP×CO

and total power output is defined by either Equation 12 for the Elbeery et al model or Equation 13 for the adjusted model. The units for Equation 15 are joules per minute (J/min).

### 2.3 Animal studies

#### 2.3.1 Surgical methods and instrumentation

The validity of the model was tested first using acute studies with adult, Dorset breed male sheep (Ovis aries) (*N* = 6, 60 ± 3 kg). Sheep were sourced from Shiloh Farm Montadales (113 Washington Pike, Avella, PA). All sheep received humane care in compliance with the “Guide for the Care and Use of Laboratory Animals” and all methods were approved by the University of Michigan Committee for the Use and Care of Animals. Anesthesia was induced with 6–9 ml/kg of propofol and maintained with 1%–3% inhaled isoflurane (Abbot Laboratories). Sheep were mechanically ventilated at all times with 100% oxygen set to a tidal volume of 10 ml/kg. The respiratory rate was adjusted to maintain an arterial PCO_2_ (PaCO_2_) between 35 and 45 mmHg. Lastly, a carotid arterial line and left jugular venous line were placed and then connected to fluid coupled pressure transducers (ICU Medical, Inc.) for animal management.

A left thoracotomy was performed, and a 24 mm perivascular flow probe (24AX, Transonic Systems, Inc.) was placed around the main pulmonary artery to measure cardiac output (CO) continuously with a T400 Flowmeter (Transonic Systems). Umbilical tape was passed around the main pulmonary artery and a Rummel tourniquet was used to increase pulmonary resistance when needed. Right coronary artery dissection was then performed by retracting the right atrium laterally to expose the atrioventricular groove. Blunt dissection with electrocauterization was performed to isolate a small section of the right coronary artery and place a 4‐mm perivascular flow probe (4AX, Transonic Systems, Inc.) to measure right coronary artery flow rate continuously (T400 Flowmeter, Transonic Systems).

Proximal PA and RV pressures were measured continuously via 14G angiocatheters (Becton, Dickinson and Company.) connected to fluid coupled pressure transducers (ICU Medical, Inc.). Lastly, an 8F pulmonary artery catheter (model 777F8, Edwards Lifesciences) was introduced into the right jugular vein and its tip placed in the pulmonary artery distal to the flow probe and umbilical tape. An ECG lead was placed on each of the sheep's legs and attached to a patient monitor (Marquette Solar). The monitor was then interfaced with the Vigilance CEDV monitor (Edwards Lifesciences) to measure heart rate (HR), right ventricular ejection fraction (EF), and end‐diastolic volume (RV_EDV_).

#### 2.3.2 Animal experimental methods

A baseline data set was taken after completing instrumentation. Proximal PA, RV, and central venous pressures and PA flow rate were acquired digitally at 250 Hz for 7 seconds using LabVIEW (National Instruments). All other data, specifically HR, RV, end‐diastolic volume, arterial saturation, and hemoglobin concentration, were hand recorded. Thereafter, RV afterload and oxygen saturation to the RV were varied. A hypoxic condition was induced by lowering the inspired oxygen fraction, FIO_2_, and thus arterial oxyhemoglobin saturation (*S*_a_). The afterload was increased by adjusting the pulmonary blood flow zeroth harmonic impedance modulus, *Z*_0_, using a Rummel tourniquet on the main PA. This index has been a preferred index for afterload by some physiologists because, unlike mPAP alone, it continues to rise as pulmonary vascular resistance (PVR) is increased to very high levels and, unlike PVR, it includes the load placed on the RV by left atrial pressure. Experimental groups were defined by the change in *Z*_0_ applied over baseline values, Δ*Z*_0_, and *S*_a_. The following four groups were thus examined; (1) Normal afterload, normoxia (*n* = 5): Δ*Z*_0_ = 0 mmHg/(L/min), *S*_a_ = 100%; (2) High afterload, normoxia (*n* = 6): Δ*Z*_0_ = 4 mmHg/(L/min), *S*_a_ = 100%; (3) Normal afterload, hypoxia (*n* = 6): Δ*Z*_0_ = 0 mmHg/(L/min), *S*_a_ = 75%; and (4) High afterload, hypoxia (*n* = 6): Δ*Z*_0_ = 4 mmHg/(L/min), *S*_a_ = 75%. Each condition was maintained for 4 hours. All data were taken every 40 minutes as at baseline. Both *S*_a_ and *Z*_0_ were assessed every 20 minutes and adjusted as needed to maintain their target values. After 4 hours, the sheep were euthanized with 90–150 mg/kg of IV pentobarbital (Fatal‐Plus, Vortech Pharmaceuticals).

### 2.4 Clinical studies

Through collaboration with the Pulmonary Hypertension Group at the Vrije Universiteit (VU) University Medical Center in Amsterdam, the Netherlands, RV hemodynamic and metabolic data for 15 idiopathic pulmonary artery hypertension (IPAH) patients were analyzed. Of the 15 patients (aged 26–71 years), 14 were female and 1 was male. The protocol was approved by the Medical Ethics Review Committee of VU University Medical Center. Each patient gave written informed consent before the study. Each patient was grouped according to the New York Heart Association Classification (NYHA). RV hemodynamic data were obtained through cardiac Magnetic Resonance Imaging (MRI), cardiac Positron Emission Tomography (PET), and right heart catheterization. Details on calculation of oxygen extraction fraction are provided in Wong, Ruiter, et al. (2011). Briefly, the entire acquisition time for PET imaging was 10 minutes, using 40 frames. Oxygen extraction fractions were determined from volume‐weighted average time‐activity curves of the identified right ventricular wall region, identified through standard methods of a C^15^O‐PET image and cardiac MRI. Pulmonary arterial pressure, right ventricular pressure, and ECG data were recorded simultaneously for most patients (*n* = 11) and asynchronously for those remaining (*n* = 4) during baseline in a supine position at a sampling rate of 1 kHz for a period of 5 minutes. Values are ensemble averaged per heartbeat over five heartbeats. For hemodynamics values and volumes, ensemble averages were calculated over five heartbeats, extracted starting from the 2 minute mark from a total 5 minute acquisition time. A summary of all provided measurements are given in Results (Table 2), below. Further details of the full clinical study can be found in Wong, Ruiter, et al. (2011).

**TABLE 2:** Summary of hemodynamics and demographics for clinical studies. Average ± standard deviation are reported

	NYHA II ( *n* = 8)	NYHA III ( *n* = 4)	NYHA IV ( *n* = 3)
Mean PA pressure (mmHg) MPAP	47.4 ± 11.7	58.8 ± 13.3	65.3 ± 22.9
Cardiac output (L/min) CO	5.6 ± 0.9	4.2 ± 1.1	3.5 ± 1.4
Ejection fraction EF	46 ± 13.3	31 ± 3.5	19 ± 4.4
Right coronary mean blood flow (ml/min) RC MBF	47.5 ± 15.5	64.6 ± 20.8	55.2 ± 12.6
Oxygen extraction fraction OEF	0.6 ± 0.15	0.7 ± 0.16	0.9 ± 0.08
RV free wall end diastolic mass (g)	72.5 ± 22.7	85.1 ± 19.7	88.1 ± 12.9
Age	43.6 ± 14.2	48.3 ± 16.8	42.0 ± 10.8
Sex (female/male)	7/1	4/0	3/0

### 2.5 Statistical analysis

To validate both models (Elbeery et al's model and the adjusted model), the coefficient of determination (*R* ^2^) was measured between the RV O_2_ consumption and RV total power output for all clinical data; for sheep, data were analyzed at baseline and after 4 hours (when hemodynamics were considered stable). Statistical significance (*p*‐value) of each model's coefficient of determination and the 95% confidence intervals were calculated. The 95% confidence interval for each regression coefficient and *y*‐intercept term were also determined. The regression coefficient is expected to reflect the theoretical efficiency of oxidative phosphorylation (20.2 J/ml O_2_) and the *y*‐intercept term is expected to be nonsignificant, reflecting the negligible contribution of anaerobic sources to total power (i.e., power output in the absence of oxygen).

To demonstrate that poor RV efficiency is related to a compensatory increase in oxygen consumption rate, the coefficient of determination between these two variables and its statistical significance is measured. RV efficiency is hypothesized to be negatively related to oxygen consumption rate (i.e., high RV efficiency allows for low RV oxygen consumption and vice versa).

To demonstrate that inefficient oxygen use in PAH is determined largely in part by demands outside of useful stroke work, average and standard error of the mean of percent contribution of isovolumic power, useful stroke power, and pulsatile stroke work to total work are reported by class for NYHA Class II and Class III/IV patients.

Finally, to demonstrate that poor RV efficiency and a compensatory need for high oxygen consumption are indicative of poor heart function, a MANOVA analysis was used to show statistically significant differences between NYHA Class II and III/IV patients in RV efficiency from both the Elbeery et al and the adjusted model as well as significant differences in oxygen consumption rate. NYHA Class III and IV patients were combined to ensure sufficient statistical power. In terms of disease severity, both NYHA Class III and IV are considered to have significant limitations in functional capacity versus NYHA Class II, making the grouping appropriate (Dolgin et al., 1994). Given a null hypothesis of no difference in these three metrics between low and high NYHA class, the alternative hypothesis is that higher NYHA class (more severe disease state) would have lower RV efficiency as calculated by both models and higher RV oxygen consumption rates. *Post hoc* analyses with Bonferroni correction were used to determine specifically which dependent variables (mechanical efficiency from the Elbeery et al model, mechanical efficiency from the adjusted model, and/or oxygen consumption rate) were significantly different between Class II and III/IV.

## 3 RESULTS

**FIGURE 1:**
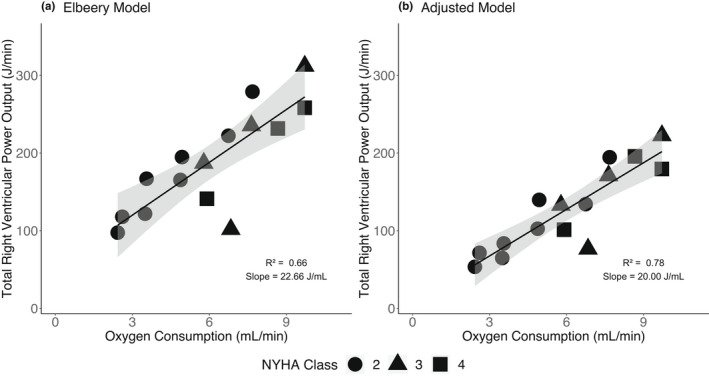
Clinical studies, relationship between right ventricular total power output and right ventricular O_2_ consumption. (a) Elbeery et al. model of total right ventricular power output. Linear regression results: Total right ventricular power output = 22.66 × Myocardial Oxygen Consumption + 52.02. The *p*‐value for *y*‐intercept = 0.09, *p*‐value for slope = 2.07 × 10^−4^. (b) Adjusted model of total right ventricular output. Marker type shows patient's disease severity (NYHA class). Linear regression results: Total right ventricular power output = 20.00 × Myocardial Oxygen Consumption + 7.48. The *p*‐value for *y*‐intercept = 0.70, *p*‐value for slope = 1.36 × 10^−5^. Bands show 95% confidence interval of the linear fit

Figure 1 shows the linear relationship between O_2_ consumption (ml/min) and right ventricular total power output (J/min) for clinical studies using both the Elbeery et al model (Figure 1a) and the newly proposed model (Figure 1b). As shown in Figure 1, the adjusted model had a higher coefficient of determination (Elbeery et al model *R* ^2^ = 0.66 vs. adjusted model *R* ^2^ = 0.78). The slope for the adjusted model (20.0 J/ml O_2_) was slightly numerically closer to the theoretical ratio of oxygen to energy output (20.2 J/ml O_2_) than the Elbeery et al model (22.66 J/ml O_2_). The *y*‐intercept for both models, a measurement of power output in the absence of O_2_, was not significantly different from zero in either model, but was reduced in the adjusted model (Elbeery et al model *y*‐intercept = 52.02, *p* = 0.09 vs. adjusted model *y*‐intercept = 7.48, *p* = 0.70).

**FIGURE 2:**
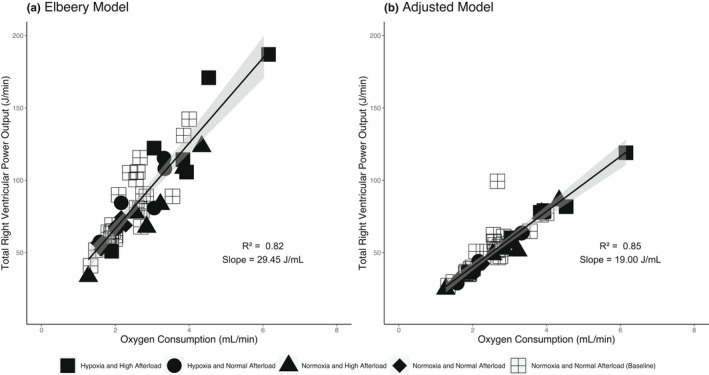
Animal Studies, Relationship Between Total Right Ventricular Power Output and Right Ventricular Oxygen Consumption. (a) Elbeery et al model of total right ventricular power output. Linear regression results: Total right ventricular power output = 29.45 × Myocardial Oxygen Consumption + 7.83. The *p*‐value for *y*‐intercept = 0.19, *p*‐value for slope = 2.07 × 10^−16^. (b) Adjusted model of total right ventricular output. Linear regression results: Total right ventricular power output = 19.00 × Myocardial Oxygen Consumption + 2.48. The *p*‐value for *y*‐intercept = 0.47, *p*‐value for slope <2.00 × 10^−16^. Marker type shows the induced condition for the animal (hypoxic and/or high afterload). Bands show 95% confidence interval of the linear fit

Figure 2 shows the linear relationship between O_2_ Consumption and right ventricular total power output for the animal studies using Elbeery et al’s model (Figure 2a) and the adjusted model (Figure 2b). The adjusted model showed a slightly higher coefficient of determination than the Elbeery et al model (Elbeery et al model *R* ^2^ = 0.82 vs. adjusted model *R* ^2^ = 0.85). Again, the slope for the adjusted model (19.00 J/ml O_2_) is numerically closer to the theoretical ratio of oxygen to energy output (20.2 J/ml O_2_) than the Elbeery et al model (29.45 J/ml O_2_). Both models did not have a significant *y*‐intercept when applied to the animal study data and again the adjusted model provided a smaller estimate of the *y*‐intercept (Elbeery et al model *y*‐intercept = 7.83, *p* = 0.19 vs. adjusted model *y*‐intercept = 2.48, *p* = 0.47).

**FIGURE 3:**
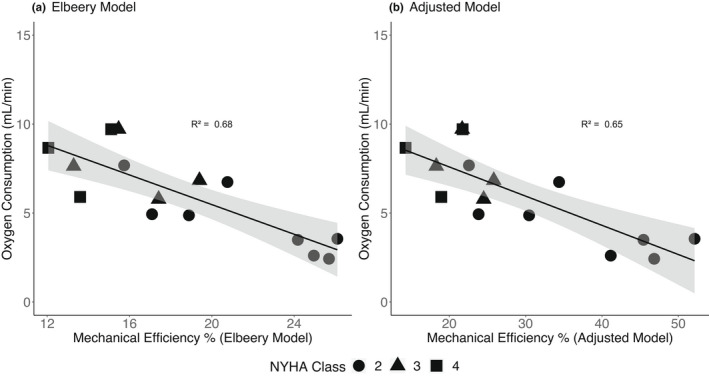
Linear relationship between right ventricular efficiency and right ventricular oxygen consumption rate. (a) Correlation performed when RV efficiency is calculated using the *Elbeery* et al model (Equation 12). (b) Correlation when RV efficiency is calculated using the adjusted model (Equation 13). Bands show 95% confidence interval of the linear fit

Figure 3 shows the correlation between RV efficiency (%) and oxygen consumption rate. The correlation between RV efficiency and oxygen consumption rate was significant for both models (*p* < 0.001). Patients of NYHA Class II were relatively clustered towards having high RV efficiency and low RV oxygen consumption rates, while NYHA Class III/IV were clustered towards having low RV efficiency and high RV oxygen consumption rates.

The average values and standard error of the mean for the percent contribution of isovolumic power, useful stroke power, and pulsatile stroke power are provided for NYHA II and III/IV are given in Table 3. Under the Elbeery et al model, differences in isovolumic work between NYHA II and III/IV compose the largest difference (78.6% vs. 68.0%), with a slight difference as well in pulsatile stroke work. However, in the adjusted model, the differences in pulsatile stroke work are negligible and the differences in isovolumic work are even more substantial (70.6% vs. 52.7%).

**TABLE 3:** Relative contributions of different forms of right ventricular power as a percentage of total right ventricular power. Mean ± standard error of the mean

Model	Type of power	NYHA II ( *n* = 8)	NYHA III/IV ( *n* = 7)
Adjusted model	Isovolumic power (J/min)	52.70 ± 5.51%	70.68 ± 2.68%
	Pulsatile stroke power (J/min)	7.81 ± 1.26%	7.03 ± 0.81%
	Useful stroke power (J/min)	37.10 ± 3.88%	20.8 ± 1.48%
Elbeery model	Isovolumic power (J/min)	68.00 ± 2.18%	78.60 ± 1.77%
	Pulsatile stroke power (J/min)	9.78 ± 1.27%	6.22 ± 1.15%
	Useful stroke power (J/min)	21.68 ± 1.45%	15.19 ± 0.96%

**FIGURE 4:**
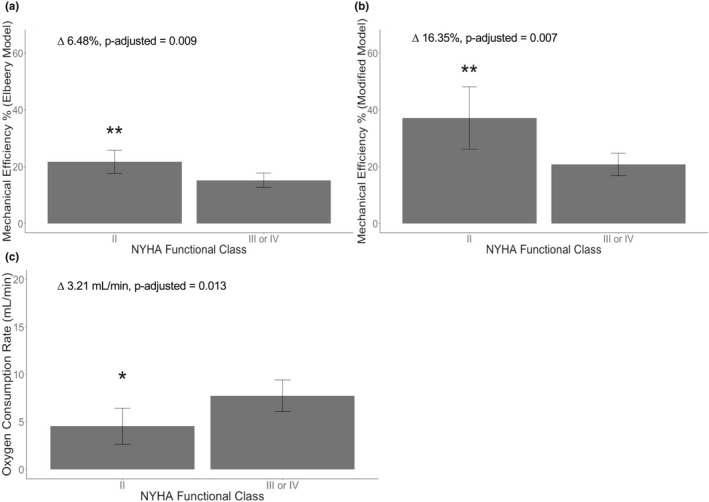
Comparison of RV efficiency and RV oxygen consumption as they relate to disease severity. Statistical significance determined by MANOVA with post hoc Bonferroni correction for multiple comparisons. (a) Difference in average RV efficiency as determined by *Elbeery* et al model (Equation 12). (b) Difference in average RV efficiency, as determined by adjusted model (Equation 13). (c) Difference in average RV oxygen consumption rate. Overall MANOVA *p*‐value = 0.013

The MANOVA analysis, with Bonferroni correction for multiple comparisons, revealed that the RV efficiency as calculated by the adjusted model and Elbeery et al model, and RV oxygen consumption rate all differed significantly between NYHA II versus NYHA III/IV patients (*p* = 0.013, Figure 4).

## 4 DISCUSSION

Two cardiac energetic models are presented that directly couple right ventricular power output and right ventricular myocardial oxygen consumption rate. Both models are novel in their application to the right ventricle under high afterload (i.e., pulmonary arterial hypertension). Adjustments made to the original model published in Elbeery et al. (1995) improved correlation strength of this coupling when applied to both sheep and human RV data, while also bringing the slope relating RV power and oxygen consumption closer to its theoretical value (20.2 joule per ml O_2_). Both models demonstrated that the estimations of total power explain 66%–78% of the variance, based on *R* ^2^, in oxygen consumption for clinical studies, and 82%–85% of the variance in oxygen consumption for animal studies. The first publication on RV energetic inefficiency related to PAH, Wong, Ruiter, et al. (2011), initially cited four other explanations for RV energetic inefficiency, including “tricuspid regurgitation, septal bowing, asynchronous activation, and/or diastolic dysfunction”, but did not identify a large, significant correlation between these effects and overall RV oxygen consumption. Our model demonstrates that, in fact, the majority of right ventricular energetic inefficiency can be explained by hemodynamics and ventricular volumes, while these other explanations might compose a smaller percentage of the variance (approximately 20%). Both models had nonsignificant *y*‐intercept terms, with the adjusted model reducing the value further, agreeing more closely with the relatively small contribution of anaerobic energy production (<5%) seen in typical human or sheep myocardial tissue.

Right ventricular efficiency, as shown by the results of the MANOVA, has a clear relationship with disease severity, with more severe right heart failure symptoms corresponding with lower RV efficiency and higher myocardial oxygen consumption. The more energy that is required to raise the end‐diastolic volume to the pulmonary arterial pressure, that is, right ventricular isovolumic work, the less efficient the RV becomes. The percentage of total power expended towards pulsatile stroke work was roughly equal for patients of low (NYHA Class II) and high disease (NYHA Class III/IV) severity, but the percentage of total power expended towards isovolumic work was much lower for low severity patients versus high severity patients (adjusted model estimates 52% vs. 75%, respectively). As raising the end‐diastolic volume to the mean PA pressure is a necessary precursor to RV ejection, this initial energy consumption limits the total energy available for stroke work and makes it more difficult for the RV to achieve sufficient stroke volume or cardiac output. Treatment goals should, therefore, focus on reducing isovolumic power output. Specifically, reduction of right ventricular end‐diastolic volume and mean right ventricular ejection pressure should be therapeutic targets for improving RV efficiency and decreasing required oxygen consumption. Further, patients with high isovolumic demands will be less capable of tolerating mild hypoxemia, and may be good candidates for oxygen therapy (Galiè et al., 2015).

The 2015 European Respiratory Society guidelines on treatment of PAH provide simple risk levels for single hemodynamics such as cardiac index, mean right atrial pressure, and mixed venous oxygen saturation (Galiè et al., 2015). However, the guidelines do not provide a comprehensive assessment of how the relationships between these variables translate to risk or how to combine them into a single quantitative risk score. While there have been multiple attempts to develop risk scores that depend on both hemodynamics and other clinical variables, the most commonly used and often cited scores (REVEAL, French Score, COMPERA) have significant limitations in their accuracy, as determined by receiver‐operator curve analysis (Benza et al., 2019; Galiè et al., 2019). Further, all risk scores are based on statistical associations of risk, rather than a deeper physiological understanding of why, for example, a high cardiac index (“low risk”) coupled with a high mean right atrial pressure (“high risk”) should translate to a specific total risk measure.

By contrast, the results of this energetic model show a physiologic justification for combining hemodynamic into comprehensive values that can demonstrate overall RV function (RV efficiency and oxygen consumption). This preliminary study demonstrated a significant association between disease severity and RV oxygen consumption and RV isovolumic work demands. As these values reflect a physiologically driven combination of crucial hemodynamics, they could potentially provide improvements to risk stratification. By expanding physiological knowledge of the behavior of the right ventricle under the progression of PAH, this model provides insights into the causal mechanisms of increased oxygen extraction and how high isovolumic power reduces energy available for stroke power, thereby reducing cardiac output. Evidence of the importance of improving physiological domain knowledge for development of statistical or machine learning clinical risk models is well‐documented (Roe et al., 2020; Sarma et al., 2020).

Further, in the search for novel PAH treatment, right ventricular energetics must be considered. Failure to consider them can have disastrous consequences. For example, efforts to improve RV contractility for PAH patients through certain inotropic drugs such as dobutamine have disadvantages specifically because they increase heart rate while not reducing preload or afterload, thereby reducing oxygen extraction reserve further and metabolically straining cardiomyocytes (Groeneveldt et al., 2019). In contrast, calcium channel blockers, such as diltiazem, slow cardiac conduction, and contractility in vasoreactive patients, reducing RV work demands and providing improved oxygen consumption reserve (Galiè et al., 2015). Further, diuretics reduce RV end‐diastolic volume, allowing for energetically favorable improvements in isovolumic power. An analysis of how pharmaceutical treatments prescribed for PAH may, in isolation or in tandem, reduce RV energetic strain could help explain observed improvements in clinical outcomes that cannot be explained by any single hemodynamic variable alone.

Novel proposed treatments for severe PAH, such as extracorporeal membrane oxygenation (ECMO), also must consider how to optimize RV oxygen delivery and avoid increasing RV work demands (Machuca & Perrot, 2015; Rosenzweig et al., 2014). Given the number of different attachment modes for ECMO, optimization of the circuit is crucial for providing the greatest therapeutic benefit, especially in situations for the use of ECMO as a bridge‐to‐lung transplant. Restorative rest for the right ventricle and improving systemic perfusion will improve transplant outcomes and have further potential for bridge‐to‐recovery (Galiè et al., 2015; Shimony et al., 2011). This improved energetic model will provide a more accurate quantification of RV offloading than the pressure‐volume area models that are currently used (Boschetti et al., 2000; Perlman & Mockros, 2007).

This model further underscores the tenuous circumstance of living with PAH and coronary artery disease. As stated, an estimated 35% of PAH patients also suffer from coronary artery disease (Shimony et al., 2011). PAH patients are dependent on higher RV oxygen consumption when afterload is advanced, and in severe PAH, maximal dilation of the right coronary is likely required to achieve the oxygen delivery necessary to maintain sufficient cardiac output. Coronary blockage will prevent patients with CAD from achieving adequate right coronary flow and will result in reduced cardiac output compared to patients with higher coronary flow, even given a similar afterload. For these patients, adequate monitoring and management of blockage is especially crucial. Angiography may be appropriate to explain low cardiac output in situations where neither RV EDV nor RV ejection pressure is exceedingly high. Further, given the greater energetic demands of the RV, these patients may require a lower threshold for percent blockage of the right coronary to become candidates for bypass.

Ultimately, for all PAH patients with progression in afterload, the compensatory increase in oxygen consumption will eventually hit a ceiling, as the right coronary artery can only dilate so far and deliver so much oxygen. This is especially true due to limitations in right coronary dilatation and flow caused by increased RV wall tension and increased right atrial pressure. Further increases in mean RV ejection pressure, venous congestion, and decreases in arterial oxygen saturation would thus eventually lead to RV failure. The ability to quantify RV oxygen consumption reserve (difference between maximal RV oxygen consumption and minimal RV oxygen consumption at rest) could provide a means of predicting how close a patient is to entering right heart failure. As oxygen consumption and RV efficiency are related, measurements of peak RV mechanical efficiency during exercise could also provide insight on how well a patient will be able to tolerate physical stress. Future work on this model will, therefore, include investigating if RV oxygen consumption reserve or mechanical efficiency reserve is indicative of survival or a patient's functional capacity (i.e., 6‐minute walk distance).

Finally, this model demonstrated that anaerobic energy sources for the RV myocardium of PAH patients were negligible, which disagrees somewhat with current thinking on this topic (Ryan & Archer, 2014). There is evidence that a metabolic switch occurs in hypertrophied myocardial tissue where anaerobic energy sources become more heavily favored, but the relative degree to which it contributes to the overall RV energy supply has not been thoroughly studied. However, both models determined that this component of RV metabolism is negligible, as there was no significant RV power output in the absence of oxygen (*y*‐intercept). In this model, it would be expected that more severe patients would experience higher levels of aerobic glycolysis, translating to a lower total power output than expected given the relative inefficiency of glycolysis versus oxidative phosphorylation. However, as shown in Wong et al's original publication, there was no significant difference in RV myocardial glucose consumption rate between NYHA II and NYHA III patients (Wong, Ruiter, et al., 2011). This suggests that inefficient oxygen use in NYHA III/IV patients is not primarily due to a greater utilization of aerobic glycolysis. Recent results of treatment of PAH with dichloroacetate intended to reduce mitochondrial decoupling and rate of aerobic glycolysis have demonstrated significant results only in patients with genetic susceptibility (Michelakis et al., 2017). Therefore, patients with a significant metabolic switch likely represent a much smaller percentage of the overall PAH population, while the models discussed here are more widely applicable.

Both Elbeery et al's model and the adjusted model have the following limitations: (1) Neither model accounts for stroke work that does not contribute to flow into the pulmonary artery due to tricuspid valve regurgitation. As a common comorbidity of PAH, this additional stroke work may be a key component to gaining a more accurate representation of total right ventricular power output (Galiè et al., 2015; Wong, Ruiter, et al., 2011). Particular to our study, a third of all patients had moderate or severe valve regurgitation (NYHA II: *n* = 1, NYHA III: *n* = 4). This would lead to a small but systematic underestimation of total power output that, while not significant on average (roughly 0.3% of total work), which could be more significant on an individual basis (up to 30% of total work). This could potentially be a significant contributor to the high residual for one patient in particular (NYHA Class III), whose O_2_ consumption rate overestimated RV total power output in the adjusted model by approximately 45%. However, it is also possible that such outliers are due to isolated measurement error in myocardial oxygen consumption via PET imaging. (2) Neither model accounts explicitly for postsystolic isovolumic contraction, a common phenomenon in PAH that is believed to further contribute to RV energetic inefficiency (Mauritz et al., 2011). Specifically, this occurs when the RV continues to contract after the pulmonic valve as closed, prolonging the period prior to tricuspid valve opening. As echocardiography data were not available in either study, it is infeasible to more finely delineate between pulmonic valve closure and RV relaxation (i.e., period immediately following RV peak shortening). The model here inferred that RV end‐systole occurs during the trough of the first derivative of the RV pressure waveform, and this corresponded strongly with the end of the T‐wave in the simultaneously measured ECG. Therefore, the models likely capture the full period of RV contraction and would account for postsystolic isovolumic contraction, but this remains speculative. Regardless, postsystolic isovolumic contraction does not appear to contribute significantly to the error in total RV power output, as RV total power output still explains the majority of the variance in RV oxygen consumption. (3) Due to the infeasibility of PET imaging for animal studies, two values are used for estimation of oxygen extraction fraction (OEF). These two values were defined in the literature and did not produce significant error in the model, though a more precise model of OEF under different hypoxic conditions would be ideal. However, this ultimately had a negligible effect on the empirical estimation of the efficiency of oxidative phosphorylation (19.0 J/ml in the adjusted model vs. 20.2 for the theoretical value) (Martínez et al., 2017).

In conclusion, both models comprehensively describe right ventricular function under high afterload, in a fashion that is physiologically rigorous. We found assumptions made for the second, adjusted model allowed for greater simplicity in calculation of total RV power output, while maintaining accuracy in measured oxidative phosphorylation efficiency and marginally improving correlation strength between power output and RV oxygen consumption. Both models combine multiple measurements of hemodynamics and right ventricular volumes to create hypothesis‐driven biomarkers of disease severity (RV efficiency and RV isovolumic work). In translation to the clinic, RV efficiency could be an important indicator of treatment efficacy, as both models demonstrate that increases in RV efficiency allow for a decreased requirement for myocardial oxygen consumption at rest. Further studies are required to determine if RV efficiency and/or RV isovolumic work are predictors of clinical outcomes.

## CONFLICT OF INTEREST

Authors have no conflict of interest and nothing to disclose.

## AUTHOR CONTRIBUTION

Jacqueline V Scott, Tanuf U Tembulkar, and Meng‐Lin Lee contributed to interpretations of data, statistical analysis, manuscript preparation, and editing. Bradley T Faliks and Kelly L Koch contributed to experimental design, data collection for animal studies, interpretations of data, manuscript preparation, and editing. Anton Vonk‐Nordegraaf contributed to experimental design and data collection of clinical studies, manuscript preparation, interpretations of data, manuscript preparation, and editing. Keith E Cook contributed to experimental design for animal studies, interpretations of data, manuscript preparation, and editing.

## Supporting information



Supplementary Material
