# Supplementary material for: Right ventricular myocardial energetic model for evaluating right heart function in pulmonary arterial hypertension
Source: Physiol Rep. 2022 May 18;10(10):e15136. doi: 10.14814/phy2.15136 (PMC9115705; doi:10.14814/phy2.15136)
Supplement: Supplementary file 1 — Supplementary Material [file PHY2-10-e15136-s001.pdf]

Title: Right Ventricular Myocardial Energetic Model for Evaluating Right Heart Function in Pulmonary Arterial Hypertension, Online Data Supplement

Authors: Jacqueline V Scott, MSc[1], Tanuf U Tembulkar, MSc[1], Meng-Lin Lee, MD, PhD[2], Bradley T Faliks, MD[3], Kelly L Koch, MD[3], Anton Vonk-Nordegraaf, MD, PhD[4], Keith E Cook, PhD[1]

Institutions:

[1] Department of Biomedical Engineering, Carnegie Mellon University, Pittsburgh, PA

[2] Division of Cardiovascular Surgery, Department of Surgery, Cathay General Hospital, Taipei, Taiwan

[3] Department of Surgery, University of Michigan, Ann Arbor, Michigan

[3] Department of Pulmonary Medicine, Amsterdam University Medical Centers, Vrije Universiteit Amsterdam, Amsterdam, the Netherlands

Right Ventricular Ejection Time Interval Algorithms

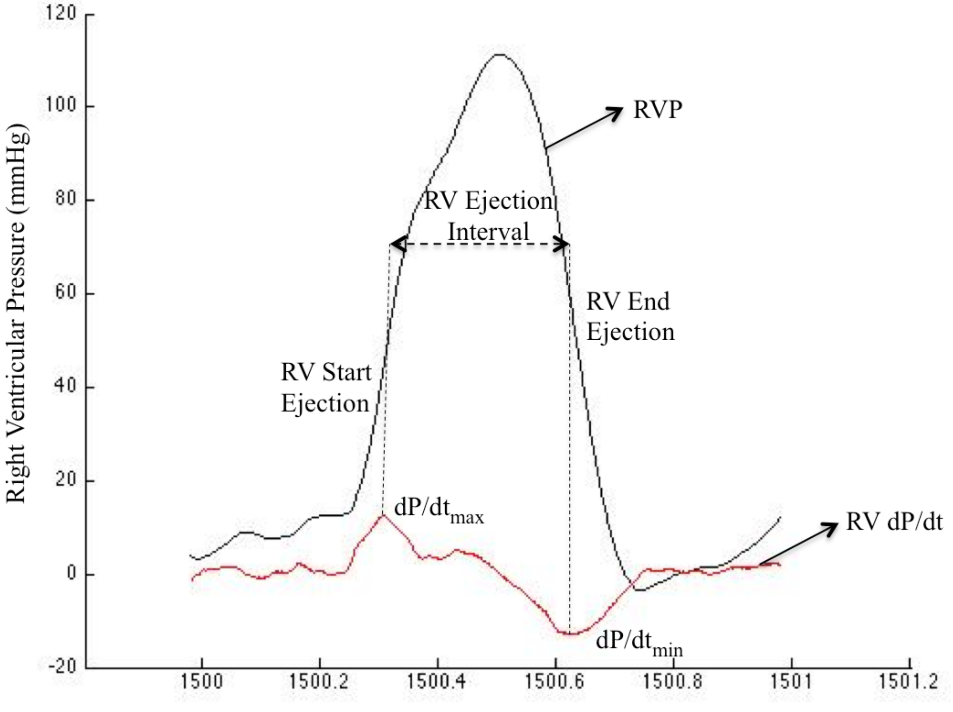

Figure E1. Beat-by-Beat RV Pressure Derivative Algorithm to Calculate RV Ejection Pressure

To determine RV ejection pressure, a beat-by-beat RV pressure waveform algorithm was used that analyzed the first derivative to determine the period of ejection. This algorithm was based on the hypothesis that maximal positive rate of change in RV pressure (dP/dt_{max}) corresponds to the opening of the pulmonic valve and thus indicates the beginning of RV ejection **(Fig. E1)**. Similarly, the maximal negative rate of change in RV pressure (dP/dt_{min}) corresponds to the closing of the pulmonic valve and indicates the end of RV ejection **(Fig. E1)**. The period between successive dP/dt_{max} and dP/dt_{min} is the RV ejection interval for a single heartbeat **(Fig. E1)**. This algorithm was implemented in MATLAB in the following way:

1. To eliminate noise from the RVP waveform, a 40-point moving average filter was applied

2. RV dP/dt was calculated using arithmetic differentiation wherein dP/dt(i-1) = RVP(i) – RVP(i-1) and dt = 0.001s (1000Hz)
3. A local peak detection algorithm was used to determine the time indices at which dP/dt_{max} (beginning of RV ejection) and dP/dt_{min} (end of RV ejection) occurred. Maxima and minima that did not form a full heartbeat were excluded.
4. The RV ejection time interval was determined for each heartbeat
5. The RV ejection pressure (RVEP) values that corresponded to the ejection time interval were obtained and averaged to arrive at the mean RVEP for a single heartbeat
6. Finally, the mean RVEPs for all the beats were averaged to obtain the mean RVEP for each patient.

For PAP and RVP waveforms that were recorded simultaneously, the ejection time interval obtained for each beat from the RVEP Algorithm is also the time period (T) from **Eq. (7)** in the main body, over which PA pressure and flow are integrated to calculate stroke power. For asynchronous recordings, the RVEP algorithm is used only to calculate mean RVEP. An alternative method, described below, is used to determine RV ejection time interval for each heartbeat. This is done to obtain the ejection time interval in terms of PA pressure time indices for stroke power integration calculation.

Method for Calculating RV Ejection Time Interval Using the Second Derivative of PA Pressure

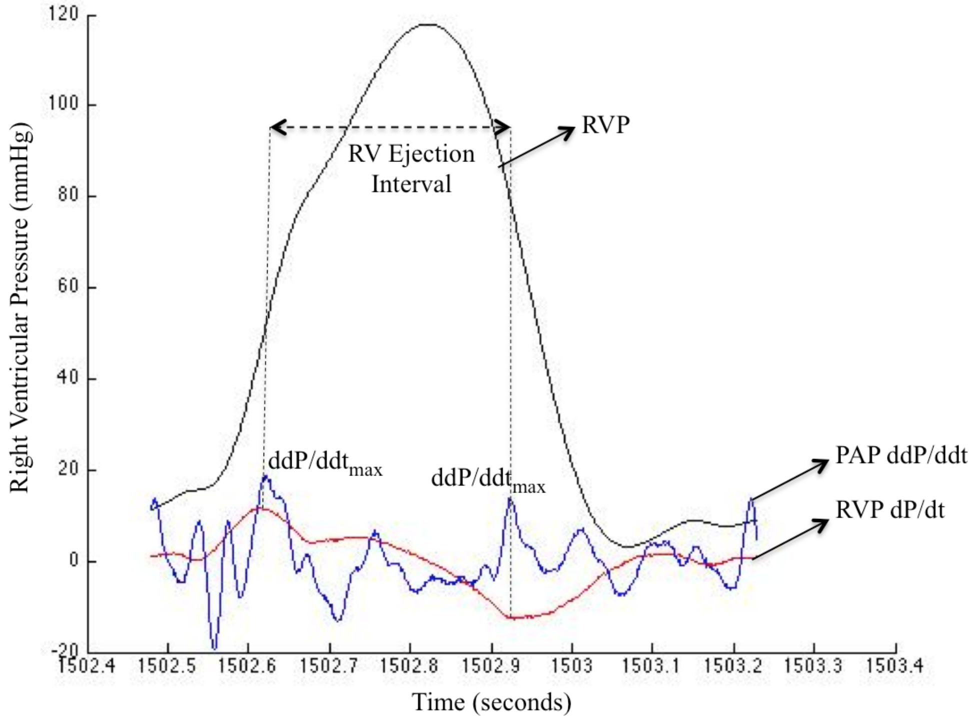

Figure E2. Beat-by-Beat PA Pressure Waveform Second Derivative Algorithm to Calculate Ejection Time Interval

The second derivative maxima of pulmonary artery pressure (ddP/ddt_{max}) indicate opening and closing of the pulmonary valve and can therefore be used to ascertain RV ejection time interval. A local peak detection algorithm was used to determine the time indices (corresponding to the PA pressure waveform) where ddP/ddt maxima occurred **(Fig. E2)**. RV ejection time interval for a single heartbeat was calculated as $t(ddP/ddt_{max}(i)) - t(ddP/ddt_{max}(i-1))$. To validate this method, the PAP second derivative algorithm was used to calculate ejection time interval and the corresponding mean RV ejection pressure in addition to the RV first derivative algorithm in patients whose RV and PA pressure waveforms were recorded simultaneously. The mean RV ejection pressures obtained from each method were then compared.

Right Ventricular Stroke Power

RV stroke power determined using the PA pressure waveform second derivative algorithm was highly predictive (R^2= 0.985) of that determined using the conventional RV pressure

waveform first derivative algorithm with almost a one-to-one correspondence ($y = 0.959x +1.997$). Therefore the PA pressure algorithm served as a valid alternative for determining ejection time interval (also the time period for stroke power integration) in patients whose RV and PA pressure recordings were asynchronous.
